# Supplier evaluation and categorize with combine Fuzzy Dematel and Fuzzy Inference System

**DOI:** 10.1016/j.dib.2018.03.077

**Published:** 2018-03-31

**Authors:** Elahe Ashtarinezhad, Amir Homayoon Sarfaraz, Mehrzad Navabakhsh

**Affiliations:** Islamic Azad University, Tehran South Branch, Faculty of Industrial Engineering, Tehran, Iran

## Abstract

Nowadays, the evaluation of the suppliers in order to improve the total performance of supply chain and increase the power of competitiveness, satisfaction and profitability of the company are considered important and significant issues at the organizations. The main objective of this research is to help oil and gas industry in order to evaluate and categorize the suppliers, using Fuzzy Inference System. The present research is empirical in terms of purpose and descriptive-survey in terms of data collection. Three outstanding managers of procurement department of the company under examination have been selected. With regard to the fact that, the number of identified Sub-indices to categorize the suppliers are too many in relevant literature, the Fuzzy Dematel method was used to determine the weight and importance of each of the Sub-indices suppliers. In the present paper, for evaluate and categorize the suppliers has been used from Fuzzy Inference System, with MATLAB Software.

**Specifications Table**

**Table:** 

Subject area	Industrial Engineering
More specific subject area	Suppliers-Fuzzy Inference System-Fuzzy Dematel
Type of data	Table, figure
How data was acquired	All criteria and sub-indices achieved based on the oral expertise interviews and based on the obligations of the company in oil and gas industry and also Prioritization of the sub-indices with providing questionnaires using Fuzzy Dematel. Classification and evaluation the suppliers using FIS was obtained with providing questionnaires and with help Matlab Software.
Data format	Raw, analyzed, etc.
Experimental factors	–
Experimental features	–
Data source location	Tehran, Iran
Data accessibility	Data are reported in this article

**Value of the data**

• The suppliers are a complementary component of the supply chain of an organization, with Nominating the proper suppliers can reduce the purchase costs considerably and increase the competitiveness capability of the organization.
• The managers will be able to plan to improve risk reduction and maximize the benefits of purchasing suppliers through comprehensive, integrated and applied approach to evaluate suppliers.
• Organizations will be able to use suppliers who are accessible to provide functional requirements and constant improvement in specific periods of time.
• With this research, we will be able to identify Sub-indices and effective criteria for evaluating suppliers in Oil & Gas industries.

## 1 Data

**Fig. 1:**
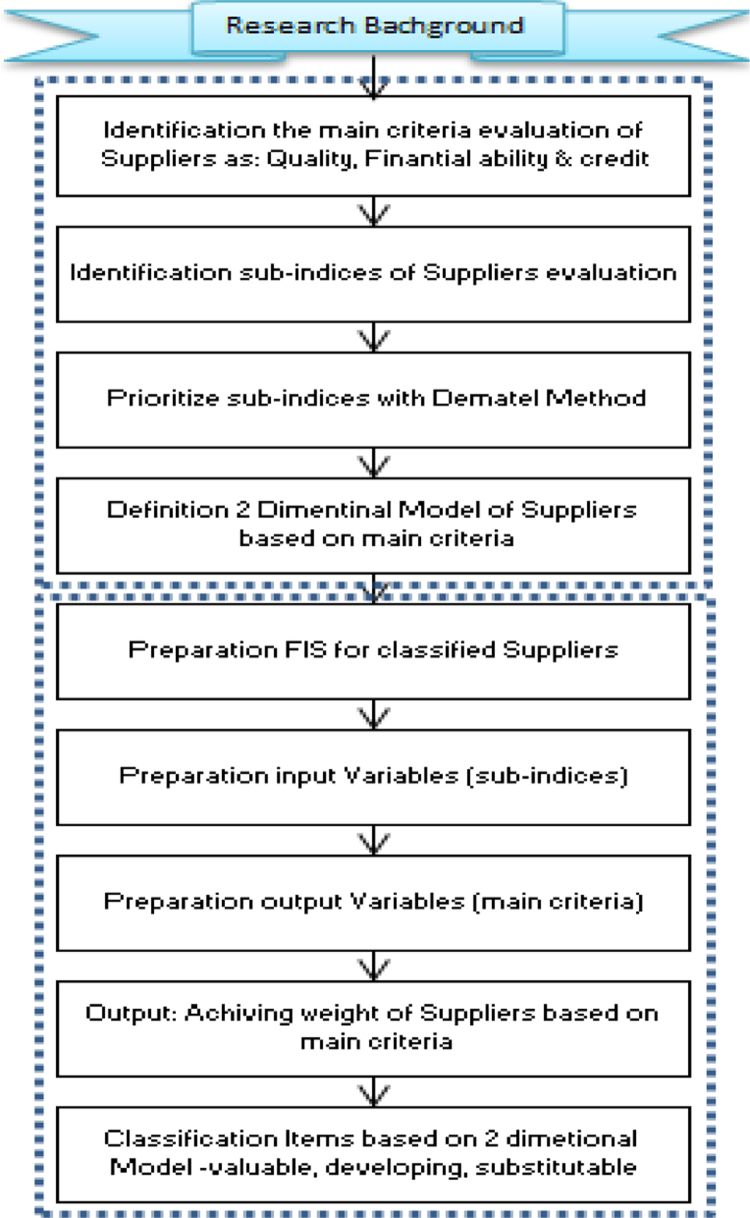
Proposed model.

The present study is empirical in terms of type and descriptive-survey in terms of data collection. The present research has been done in one of the great oil projects in 2016. Three outstanding managers of procurement department have been selected as to analyze the data. The methodology of doing this research is presented in Fig. 1.

### 1.1 Identification of the major criteria of evaluation of the suppliers

Identification of the major criteria of evaluation of the suppliers based on two-dimensional model of Olsen and Ellram [1], by the oral expertise interviews is based on the obligations of the company in oil and gas industry. Two major criteria of Quality and Financial ability and credit of company were selected as the most important criteria for the evaluation of suppliers.

### 1.2 Identification of the sub-indices evaluation of the suppliers and prioritization of them with DEMATEL Fuzzy method of

After reviewing the studies and using oral interviews, number of sub-indices was selected based on obligations of the company in oil and gas industry.

For the weights sub-indices, we used the Dematel Fuzzy Method with providing questionnaires. A paired comparison between the sub-indices is to be made in a Fuzzy manner, Table 1, Table 2 display paired comparison Matrix (it should be noted that Table 1, Table 2 are the result of the average score of three outstanding managers). After calculating the Normal matrix, weight of each of the sub-indices was determined (Table 3, Table 4) and the ones having a total weight of more than 50% were selected as the selected sub-indices, and the calculation results are represented in Table 5.

**Table 1:** Paired comparison matrix for quality sub-indices.

**Table 2:** Paired comparison matrix for financial ability and credit sub-indices.

**Table 3:** Calculation of the final weights quality sub-indices using the Dematel Fuzzy.

**Quality sub-indices**	**Weight**
History of cooperation with the company	0.09
Possessing Quality Assurance Certificate	0.13
Running major Contracts & Executive History/Background	0.13
Provide product certification	0.10
Presence in Oil & Gas Industries Approved Vendor List	0.14
Report	0.08
Project Management	0.09
Organizational structure and personnel of the company	0.10
After sales Services	0.11

**Table 4:** Calculation of the final weights Financial ability and credit sub-indices using the Dematel Fuzzy.

**Financial ability** and **credit sub-indices**	**Weight**
Presence in Oil & Gas Industries Approved Vendor List	0.145
Running major Contracts & Executive History/Background	0.127
Communication with Foreign Companies	0.127
Capacity (sales) of annual supply of goods	0.110
Opportunities for goods sale	0.107
Specification/Size Limit	0.123
Field of activity	0.083
Release the goods	0.086
warehouses	0.093

**Table 5:** Selected sub-indices.

**Row**	**Selected sub-indices related to quality**	**Row**	**Selected sub-indices related to financial ability and credit**
C1	Presence in Oil & Gas Industries Approved Vendor List	C5	Presence in Oil & Gas Industries Approved Vendor List
C2	Possessing Quality Assurance Certificate	C6	Running major Contracts & Executive History/Background
C3	Running major Contracts & Executive History/Background	C7	Communication with Foreign Companies
C4	After sales Services	C8	Specification/Size Limit

In the next step, the selected sub-indexes ($C_{i}$) will be used as input variables.

### 1.3 Definition a two-dimensional model based on major criteria

**Fig. 2:**
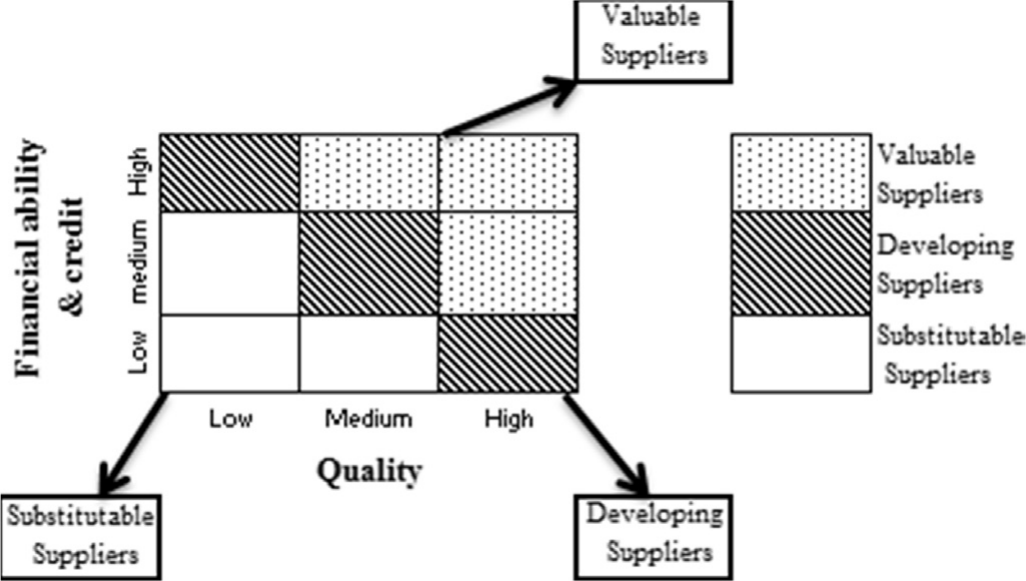
Suppliers classification model.

In this stage, suppliers are classified in terms of Quality, Financial ability and credit of company based on Olson and Ellram [1] two-dimensional model into three positions of Valuable, Developing and Substitutable Suppliers represented in Fig. 2.

### 1.4 Classification and evaluation the suppliers using Fuzzy Inference System

**Fig. 3:**
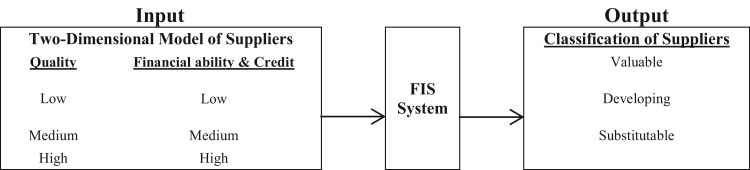
Input and output variable.

In this stage, in order to determine the Min and Max Values, the set of Fuzzy numbers in each section for all input and output variables of the interval it has been chosen based on Laura osiro et al. [2] essay and view of expert. In this research, for all input and output variables, triangular membership functions have been used (Fig. 3).

The following set of Fuzzy Numbers for each sub-indices (Input Variables) were calculated and used per criterion (Output Variables) and taking into consideration Fuzzy Logic (Table 6, Table 7, Table 8).

**Table 6:** Membership functions of criteria related to quality.

	**Low triangular**			**Medium triangular**			**High triangular**		
	L	M	H	L	M	H	L	M	H
C1	0	3.5	4	3	5.5	8	7	8	10
C2	0	3	4	3	5.5	7.5	7	9.5	10
C3	0	2.5	3	2.5	5	7	6.5	9.5	10
C4	0	2.5	3.5	3	6	7.5	7	8.5	10

**Table 7:** Membership functions of criteria related to financial ability and credit.

	**Low triangular**			**Medium triangular**			**High triangular**		
	L	M	H	L	M	H	L	M	H
C5	0	3	4	3	5.5	8	7	8.5	10
C6	0	2.5	3	2.5	5	7	6.5	9	10
C7	0	3	4	3	6.5	7.5	7	9	10
C8	0	2.5	4	3.5	5.5	7.5	7	9.5	10

**Table 8:** Membership functions of output variables.

		**Low triangular**			**Medium triangular**			**High triangular**		
		L	M	H	L	M	H	L	M	H
Quality		0	2.5	3.5	3	6	7.5	7	8.5	10
Financial ability and credit		0	3	4	3	5.5	8	7.5	8.5	10

In this section, it has been used Mamdani [3] Fuzzy Inference system with help Matlab Software.

**Fig. 4:**
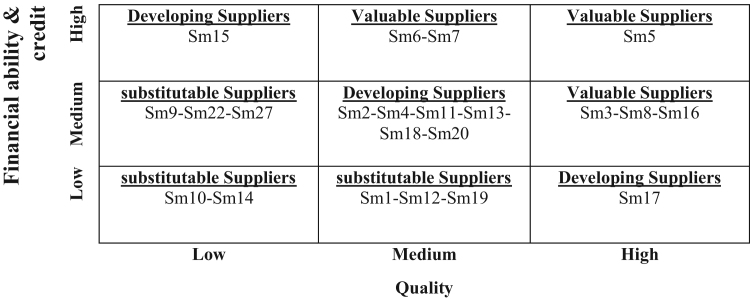
Classification of suppliers.

After obtaining average aggregation of opinions (three managers) (Table 9), each of the ($C_{i}$) insert in Matlab as Input variables and the obtained crisp numbers were calculated the performance amount of each supplier (Table 10) and finally each supplier was placed at valuable, developing and substitutable suppliers categories regarding the obtained Outputs of Fuzzy Inference of suppliers, as represented in Fig. 4.

**Table 9:** Average aggregation of opinions.

**Suppliers**	**Quality**				**Financial ability and credit**			
	C1	C2	C3	C4	C5	C6	C7	C8
**Sm1**	5.4	4.0	6.9	4.7	6.3	4.5	5.0	3.8
**Sm2**	5.5	5.8	4.0	9.0	3.8	9.8	3.4	5.3
**Sm3**	6.3	5.0	9.2	5.2	8.70	5.40	5.50	4.90
**Sm4**	4.2	5.6	6.3	4.9	7.20	4.80	6.47	4.50
**Sm5**	9.4	7.3	9.1	6.5	8.8	6.6	5.1	7.5
**Sm6**	7.9	8.5	6.8	5.3	5.2	7.2	8.1	7.8
**Sm7**	6.5	7.2	5.1	6.1	5.9	6.9	9.4	8.3
**Sm8**	7.8	6.3	5.4	4.1	5.1	4.9	5.8	6.5
**Sm9**	5.9	4.6	3.5	6.1	4.1	6.2	5.1	6.6
**Sm10**	4.0	4.5	3.2	4.6	3.5	4.5	5.4	3.4
**Sm11**	9.3	8.5	3.3	9.1	6.2	6.0	4.2	3.9
**Sm12**	9.5	8.5	3.3	9.0	4.2	8.1	4.4	3.4
**Sm13**	3.1	2.5	8.3	7.1	7.1	8.6	4.7	6.4
**Sm14**	3.4	3.2	3.9	4.2	3.7	4.8	4.3	3.1
**Sm15**	5.5	2.8	3.3	6.3	4.8	7.4	9.9	8.3
**Sm16**	7.0	5.6	7.9	8.0	5.1	5.9	3.9	5.3
**Sm17**	8.2	6.3	6.6	6.8	4.9	5.1	4.1	3.3
**Sm18**	7.1	4.4	6.9	7.1	7.1	5.0	4.4	5.5
**Sm19**	5.9	4.5	4.8	3.1	4.5	3.7	3.9	3.5
**Sm20**	7.1	8.5	7.0	5.8	6.6	5.7	7.3	6.5

**Table 10:** Obtained crisp numbers for performance of suppliers.

**Suppliers**	**Quality**		**Financial ability and credit**	
	**Out put**	**Linguistic variable**	**Out put**	**Linguistic variable**
**Sm1**	6.00	Medium	2.05	Low
**Sm2**	6.00	Medium	5.50	Medium
**Sm3**	8.87	High	5.50	Medium
**Sm4**	6.00	Medium	5.50	Medium
**Sm5**	8.80	High	8.55	High
**Sm6**	6.40	Medium	8.65	High
**Sm7**	6.00	Medium	8.60	High
**Sm8**	8.90	High	5.50	Medium
**Sm9**	3.52	Low	5.50	Medium
**Sm10**	3.06	Low	2.20	Low
**Sm11**	6.41	Medium	5.50	Medium
**Sm12**	6.53	Medium	2.20	Low
**Sm13**	6.25	Medium	5.45	Medium
**Sm14**	4.03	Low	2.15	Low
**Sm15**	3.29	Low	8.55	High
**Sm16**	8.34	High	5.50	Medium
**Sm17**	8.77	High	2.15	Low
**Sm18**	6.46	Medium	5.45	Medium
**Sm19**	6.00	Medium	2.15	Low
**Sm20**	6.37	Medium	5.50	Medium

## 2 Experimental design, materials and methods

With regard to the findings of the research, it can be stated that the suppliers are a complementary component of the supply chain of an organization, and they must be correctly selected and evaluated. First, according to the studies carried out in this field and oral expertise interviews, two major criteria of Quality and Financial ability and credit of company have been identified. Using the Fuzzy Dematel method and analyzing the causal relationships, the intensity and effectiveness of the sub-indices of each of the criteria were determined and given that the supplier's evaluation is inherently confronted with inaccurate data, the Fuzzy Inference System has been used by MATLAB software to evaluate suppliers. Finally, the suppliers with the final scores obtained in the two-dimensional model of suppliers were classified into three categories of Valuable, Developing and substitutable suppliers.
